# Data-Driven Image-Based Protocol for Brain PET Image Harmonization

**DOI:** 10.3390/s25134230

**Published:** 2025-07-07

**Authors:** Eva Štokelj, Urban Simončič, for the Alzheimer’s Disease Neuroimaging Initiative

**Affiliations:** 1 Faculty of Mathematics and Physics, University of Ljubljana, 1000 Ljubljana, Slovenia; 2 Jozef Stefan Institute, 1000 Ljubljana, Slovenia

**Keywords:** FDG-PET, image harmonization, brain imaging, multicenter studies

## Abstract

**Highlights:**

**What are the main findings?**

- A novel image-based data-driven harmonization protocol successfully estimates scanner-specific filters for brain FDG-PET, without requiring phantom scans.
- Harmonization accuracy is robust for moderate target resolutions (8 and 10 mm) but is notably reduced at higher resolutions (6 mm).

**What is the implication of the main finding?**

- This method facilitates harmonization of retrospective multicenter FDG-PET brain studies, enhancing comparability even when phantom calibration data are unavailable.
- Limitations identified at higher resolutions highlight the need for further methodological improvements, aligning harmonization strategies with recent advancements in high-resolution PET imaging.

**Abstract:**

Quantitative FDG-PET brain imaging across multiple centers is challenged by inter-scanner variability, impacting the comparability of neuroimaging data. This study proposes a data-driven image-based harmonization protocol to address these discrepancies without relying on traditional phantom scans. The protocol uses spatially normalized FDG-PET brain images to estimate scanner-specific Gaussian smoothing filters, optimizing parameters via the structural similarity index (SSIM). Validation was performed using images from cognitively normal individuals and Alzheimer’s disease patients from the Alzheimer’s Disease Neuroimaging Initiative (ADNI) database. Results demonstrated robust harmonization at moderate target resolutions (8 and 10 mm FWHM), with filter estimates consistently within 1.2 mm of phantom-derived ground truths. However, at higher resolutions (6 mm FWHM), discrepancies reached up to 3 mm, reflecting reduced accuracy. These deviations were particularly evident for high-resolution scanners like HRRT, likely due to elevated noise levels and smaller sample sizes. The presented harmonization method effectively reduces inter-scanner variability in retrospective FDG-PET studies, especially valuable when phantom scans are unavailable. Nonetheless, the current limitations at finer resolutions underline the necessity for methodological refinements to meet the demands of evolving high-resolution PET imaging technologies.

## 1. Introduction

Quantitative Positron Emission Tomography (PET) imaging, using various radiotracers, is essential for studying brain function and pathology, measuring metabolic processes, and serving as a biomarker in numerous clinical and research applications [1,2,3,4,5,6,7,8,9]. Depending on the characteristics of the radiotracer, different quantitative metrics are employed to assess tracer uptake. However, the variability in PET data acquired from different scanners and imaging centers presents a significant challenge for multicenter studies.

Several strategies have been suggested to acquire data and minimize inter-system variability in quantitative multicenter brain PET studies [10,11,12,13]. These strategies typically involve optimizing reconstruction parameters and post-reconstruction smoothing to meet specific quantitative image quality indicators, such as target image resolution (measured in mm full width at half maximum; FWHM), recovery coefficient, and signal-to-noise ratio. To determine these metrics, phantom scans with known geometries and activity distributions are usually performed and compared to digital reference objects [12,14].

A prominent example of multicenter PET brain study harmonization is the Alzheimer’s Disease Neuroimaging Initiative (ADNI) study, where Joshi et al. proposed a two-step harmonization method using the Hoffman 3D brain phantom [10]. They used a digital Hoffman phantom image smoothed with an 8 mm full width at half-maximum (FWHM) Gaussian filter as the target resolution and selected an appropriate FWHM Gaussian filter for each scanner model. This scanner-specific smoothing approach effectively reduced inter-scanner variability, although low-frequency correction was found to be ineffective. Similarly, Ikari et al. used an 8 mm FWHM image resolution as a reference level in the Japanese-ADNI (J-ADNI) study, harmonizing image resolution with a scanner-specific smoothing filter [14,15].

Another option is to employ gray matter contrast recovery (RCGM) and gray-to-white matter contrast (GMWMr) using the Hoffman 3D brain phantom for harmonization [13,16]. Verwer et al. proposed upper and lower limits for RCGM and GMWMr to harmonize image contrast, similar to the approach used for whole-body PET [13]. Despite its widespread use, the Hoffman 3D brain phantom can only simulate the distribution pattern of FDG in the brain [17]. Different phantoms may better model radioactivity distributions other than those of FDG. For instance, Hoye et al. harmonized ^11^C-raclopride brain PET images measured by HRRT (high-resolution research tomograph, Siemens) and HR+ (a standard clinical scanner, Siemens) using a 3D brain phantom [18]. Fahey et al. evaluated image uniformity, spatial resolution, and image quality of 13 PET scanners using the SNMMI CTN brain phantom, which includes a uniform section, a resolution section, and a clinical brain simulation section [19]. Ruwanpathirana et al. harmonized Aβ-PET images across scanners using a barrel phantom designed for spatial resolution matching, demonstrating improved consistency in quantitative analysis [20]. Wagatsuma et al. introduced a novel phantom tailored to tau PET imaging, addressing tracer-specific harmonization needs beyond FDG [21].

Beyond physical phantom-based harmonization, several studies have focused on reconstruction and validation strategies to enhance spatial and quantitative accuracy. Verrecchia-Ramos et al. demonstrated that incorporating PSF modeling and correcting for edge artifacts during reconstruction improved the quantitative reliability of brain PET images [22]. Omidvari et al. evaluated the NeuroEXPLORER PET scanner and highlighted the need for advanced validation protocols that extend beyond standard phantom evaluations, particularly in the context of diverse neuroimaging applications [23].

Adjusting reconstruction settings and applying additional smoothing filters based on the data from phantom images is a standard approach for PET harmonization. It falls under the “image-based” harmonization category, as the “harmonized images” need to be generated before measuring quantitative metrics in PET images. Nevertheless, harmonization can also be performed on the metrices derived from PET images. While the “image-based” harmonization generally requires scans of phantoms on each scanner to capture the scanner-specific characteristics of the images, harmonization on the metrics’ level is typically data-driven and does not require phantom scans. A representative example of harmonization on the metrics’ level is the ComBat harmonization method [24]. It was originally proposed to reduce “batch effects” in genomics [25]. ComBat directly applies to quantitative values derived from PET images, eliminating the need for additional image data processing and phantom data acquisition. Orlhac et al. provided a practical guide and outlined limitations when applying ComBat to image-derived quantitative metrics [26]. So far, the ComBat method has been used for FDG-PET harmonization in neurology [27] and oncology [28,29,30].

Traditional image-based harmonization with phantom scans is generally applicable and usually provides sufficient harmonization of PET images in multicenter studies. Thus, it might be the best option if appropriate phantom scans are available. In contrast, data-driven harmonization is applicable when appropriate phantom scans are not available (e.g., in retrospective studies) but imposes limitations on the image analysis. To avoid the use of phantom scans while mitigating the limitations imposed on image analysis, some recent studies have harmonized brain PET images based exclusively on the imaging data of study subjects. For example, Jin et al. applied empirical Gaussian smoothing to reduce differences in image sharpness between cohorts, guided by gradient-based visual assessment and demonstrated its utility in focal cortical dysplasia detection [31]. However, their approach did not attempt to quantify spatial resolution or generalize beyond the datasets used. In contrast, Carbonell et al. proposed a method to directly estimate scanner-specific resolution from patient data and validated it using matched phantom acquisitions [32]. These examples highlight the need for practical harmonization techniques that are both data-driven and applicable to retrospective studies where scanner resolution metadata or phantom data are unavailable.

In this study, we aimed to develop a data-driven image-based protocol for brain PET image harmonization. The goal is to determine the appropriate scanner-specific smoothing filters that are used to make the images across different scanners comparable in resolution. Unlike the traditional approaches that utilize phantom scans for cross-scanner calibration, our method leverages the inherent characteristics of spatially normalized PET images. The method is validated with the data from phantom scans, which provide “ground truth” for the appropriate scanner-specific smoothing filters.

## 2. Materials and Methods

### 2.1. Data-Driven Image-Based Harmonization Protocol

The harmonization protocol is designed to harmonize the resolution of the FDG-PET brain images from two sources that may have different image resolution. Images from one source (reference images) serve as a reference image resolution, which should be lower or the same as the resolution of images from another source (test images). The main task of this harmonization protocol is to find the filter parameters (XY Gaussian filter kernel and Z Gaussian filter kernel) for the test images, so that the final resolution of the test images corresponds to the resolution of the reference images.

Reference and test images are spatially and intensity normalized, so that the differences among them originate from four sources: (1) intrinsic differences in brain metabolic activity among the subjects, (2) image noise, (3) imperfect spatial normalization, and (4) different spatial resolution. Intrinsic differences in brain metabolic activity among the subjects and image noise are reduced by averaging multiple images from the same scanner. Imperfect spatial normalization further reduces spatial image resolution when the average of multiple images is calculated. If this effect is low or comparable in the reference and test image sets, the main differences between the average reference and test images are due to the different spatial resolution of the images.

The harmonization protocol aims to find the filter parameters for the test images that maximize the structural similarity index metric between the average reference image and the filtered average test image. The optimal filter parameters $\Theta *=\left(FWHM_{XY},\;FWHM_{Z}\right)$ are found by solving the following optimization problem:

$$\Theta *=arg\;\underset{\Theta }{\max}\;SSIM\left(I_{ref},\;G\left(\Theta \right)\;*\;I_{test}\right)$$

Here, *I_ref_* denotes the smoothed reference image, *I_test_* the average test image, *G*(Θ) the 3D Gaussian filter, and *SSIM* the structural similarity index metric.

### 2.2. Validation of the Harmonization Protocol

In this study, we used FDG-PET brain images obtained from the Alzheimer’s Disease Neuroimaging Initiative (ADNI) database (adni.loni.usc.edu). The ADNI was launched in 2003 as a public–private partnership led by Principal Investigator Michael W. Weiner, MD. The primary goal of ADNI has been to test whether serial magnetic resonance imaging (MRI), PET, other biological markers, and clinical and neuropsychological assessment can be combined to measure the progression of mild cognitive impairment (MCI) and early Alzheimer’s disease (AD). 

Half of the selected patients had AD diagnosis clinically confirmed at all follow-up sessions. The remaining were selected among cognitive normal (CN) subjects who were confirmed as CN at all follow-up sessions. Only images that were acquired with scanners yielding a final image resolution of 6 mm full width at half maximum (FWHM) or less were considered.

Under these criteria, 240 subjects’ images were selected randomly from the ADNI database. Images that were used were preprocessed by ADNI with co-registration, averaging and standardization of image and voxel size (they were not filtered to standard resolution 8 mm FWHM). The voxel size of the ADNI preprocessed images was 2 × 2 × 2 mm^3^, and their image matrix size was 160 × 160 × 96. Selected FDG-PET images were acquired by four different scanners. Table 1 shows selected scanner models, their effective resolution, and the number of AD and CN subjects selected for each scanner.

For the validation of the harmonization process, we repeatedly selected one scanner’s image dataset as a reference dataset and smoothed its average image to achieve a desired target resolution. The smoothing filter for the reference image was computed using the standard model assuming that the final spatial resolution is determined by the quadratic addition of the scanner resolution and the Gaussian filter kernel:

$$FWHM_{target}^{2}=FWHM_{scanner}^{2}+FWHM_{filter}^{2}$$

Solving for the filter width gives the following:

$$FWHM_{filter}^{2}=\sqrt{FWHM_{target}^{2}-FWHM_{scanner}^{2}}$$

This equation was applied separately in the XY and Z directions, using scanner-specific FWHM values from Table 1.

Each of the four scanner’s image datasets served as a reference dataset, while the other three scanners’ image datasets served as test datasets. For each reference image dataset, we did harmonization for three reference image resolutions: 6 mm, 8 mm, and 10 mm FWHM. We chose the target resolutions to represent commonly used resolutions in clinical and research FDG-PET studies, balancing practical relevance with methodological constraints.

The harmonization protocol was conducted separately for CN subjects and AD patients to check for any differences in the harmonization process’s performance. Estimated filter parameters were compared to the required filter parameters evaluated from the effective scanner resolution.

All FDG-PET images were spatially normalized using a standard PET template based on the Montreal Neurological Institute (MNI). After spatial normalization, the final image dimensions were 79 × 95 × 78 voxels, with the voxel size remaining at 2 × 2 × 2 mm^3^. Image normalization was performed using Statistical Parametric Mapping 12 (SPM12; Institute of Neurology, UCL, London, UK) software running in Matlab R2020a (MathWorks Inc., Natick, MA, USA). The harmonization protocol was implemented in Matlab R2024a (MathWorks Inc., Natick, MA, USA). The structural similarity index metric was calculated with the Matlab function ssim() and optimized with the Matlab function fmincon(). To start the optimization, we first obtain the approximate filter parameters by the grid search. Then we used this value as a starting point for the fmincon(). The lower bound for fmincon() was fixed at 0 mm, while the upper bound was adjusted according to the target resolution: 6 mm, 10 mm, and 16 mm for target resolutions of 6 mm, 8 mm, and 10 mm, respectively. These bounds were chosen to allow sufficient search space and to avoid constraining the optimizer in a way that could introduce estimation bias.

## 3. Results

**Figure 1:**
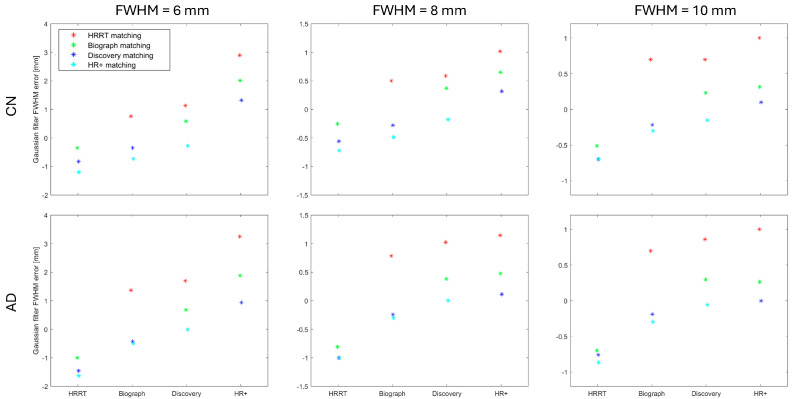
Estimated Gaussian filter FWHM error [mm] for the XY plane when the selected scanner (horizontal axis) is matched to another scanner (marker shape and color). Results are shown for three target resolutions and two image datasets: CN subjects and AD patients.

**Figure 2:**
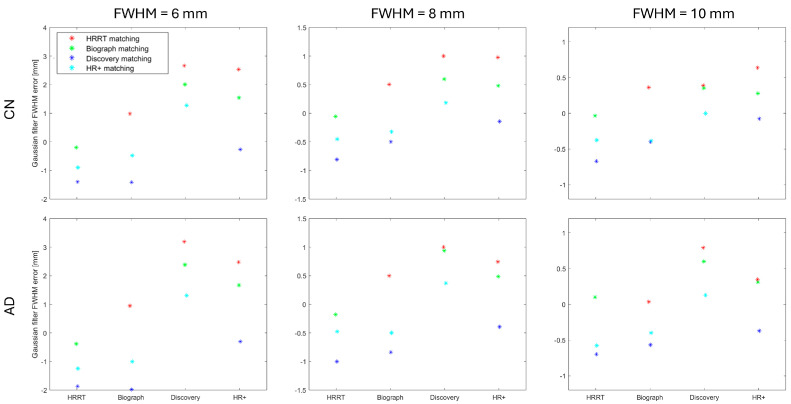
Estimated Gaussian filter FWHM error [mm] for the Z plane when the selected scanner (horizontal axis) is matched to another scanner (marker shape and color). Results are shown for three target resolutions and two image datasets: CN subjects and AD patients.

The results of our data-driven image-based harmonization protocol are presented in Figure 1 and Figure 2. These figures show the estimated Gaussian filter FWHM error [mm] when the selected scanner (named on the horizontal axis) is matched to another scanner, denoted with the marker shape and color (explained in legend).

Figure 1 presents the results for the estimated Gaussian filter FWHM for the XY plane, whereas Figure 2 is for the Z plane. Each figure includes results for all three target resolutions and two image datasets (CN subjects and AD subjects).

The data-driven image-based harmonization protocol for brain PET images shows consistent pattern for both CN and AD across all target resolutions. When the estimated Gaussian filter FWHM is lower (or higher) than the ground truth from the phantom scan, that difference is generally in the same direction for both subject groups and in all three resolutions.

The difference between the ground truth for the Gaussian filter FWHM and the estimation can be substantial when trying to achieve the 6 mm FWHM resolution. For example, the HR+ has a ground truth XY-direction Gaussian filter FWHM set at 0.3 mm, while matching to other scanners estimates its value in the range of 2–3.5 mm. A similar problem occurs when we try to find the appropriate Z-direction Gaussian filter for HR+ or Discovery. All three cases share a very small Gaussian filter FWHM ground truth.

When the image requires substantial filtration to achieve the desired resolution, the required Gaussian filter FWHM can be estimated using the proposed data-driven image-based harmonization protocol. For the 8 mm and 10 mm target resolution, where the required Gaussian filter FWHM was 5.0 mm or more, the estimated Gaussian filter FWHM was always within 1.2 mm of the true value.

**Figure 3:**
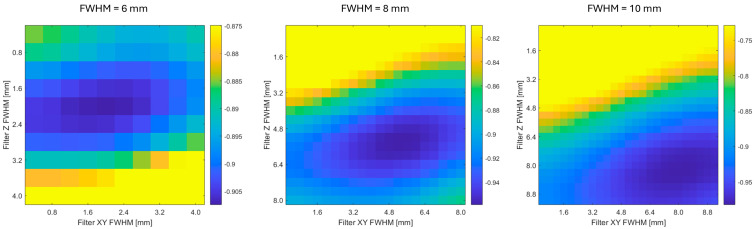
The SSIM map as a function of XY and Z Gaussian filter FWHM parameters for one representative scanner pairing. The unimodal shape confirms the stability of the optimization procedure and supports the reliability of the estimated filter parameters. Note that the colorbar scale is saturated at higher values to better visualize the valley around the minimum.

To assess the robustness and reliability of the optimization procedure, we evaluated the SSIM metric across a 2D grid of XY and Z Gaussian filter FWHM values. Figure 3 shows an example of the resulting SSIM surface, computed for one representative scanner pairing and target resolution. The surface is unimodal and exhibits a well-defined global minimum, confirming that the optimization landscape is smooth and free of local extrema. The shape of the SSIM map also illustrates the sensitivity of the SSIM metric to changes in filter parameter for each direction and provides a qualitative estimate of parameter uncertainty and interdependence.

**Figure 4:**
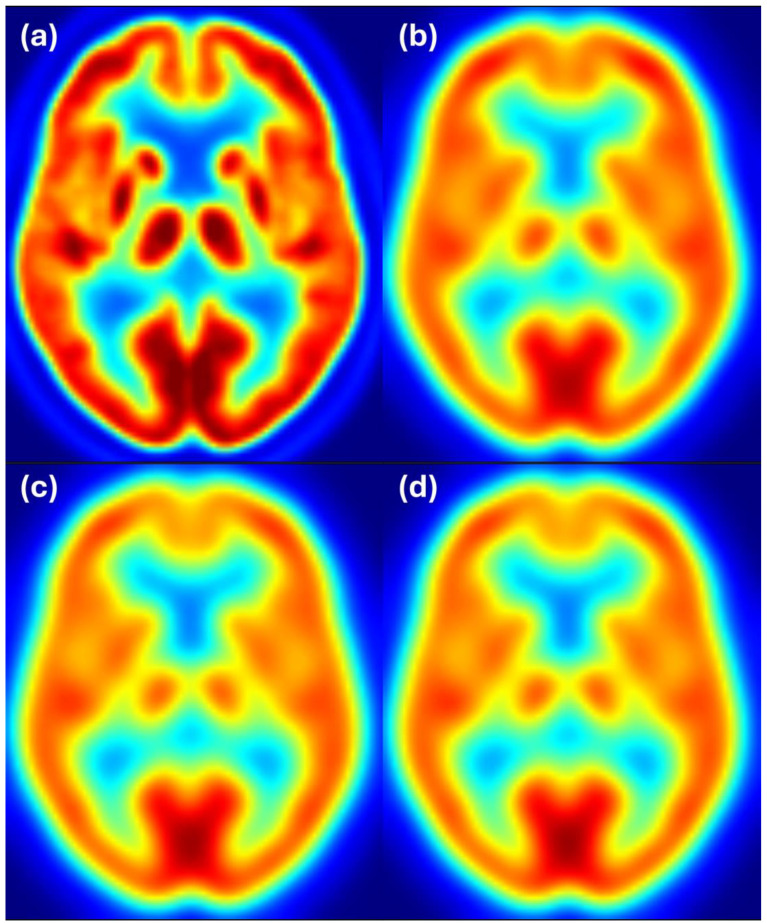
Harmonization results for the Discovery scanner matching the HR+ images filtered to 8 mm resolution. Image (**a**) shows the unfiltered Discovery image, (**b**) shows the HR+ image at the target resolution of 8 mm, (**c**) shows the Discovery image after filtering with the estimated filter, and (**d**) shows the Discovery image filtered to 8 mm resolution. Color scale indicates FDG uptake levels: red for high, green/yellow for medium, and blue for low uptake.

To complement the quantitative results, we provide visual examples of harmonization outcomes for two representative scanner combinations. Figure 4 shows axial slices for 8 mm target resolution for Discovery matching HR+ resolution. It shows that the Discovery image after filtering with the estimated filter is almost the same as the Discovery image filtered to 8 mm resolution. Both images also closely resemble the reference image from the HR+ scanner with 8 mm resolution.

**Figure 5:**
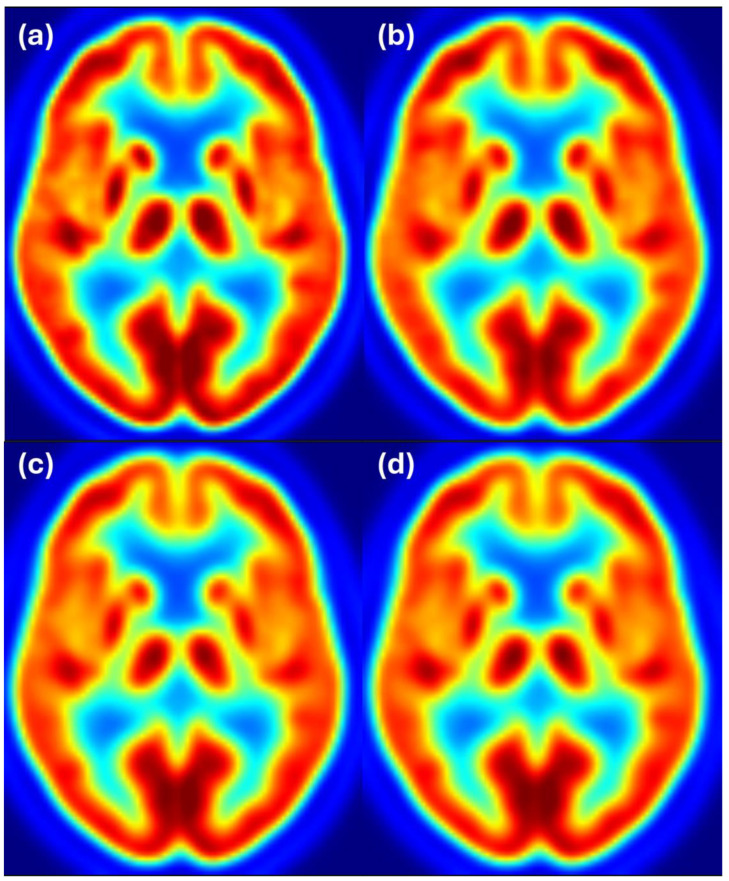
Harmonization results for the Discovery scanner matching the HR+ images filtered to 6 mm resolution. Image (**a**) shows the unfiltered Discovery image, (**b**) shows the HR+ image at the target resolution of 6 mm, (**c**) shows the Discovery image after filtering with the estimated filter, and (**d**) shows the Discovery image filtered to 6 mm resolution. Color scale indicates FDG uptake levels: red for high, green/yellow for medium, and blue for low uptake.

Figure 5 shows axial slices for 6 mm target resolution for Discovery matching HR+ resolution. Here we also see that the Discovery image after filtering with the estimated filter is almost the same as the Discovery image filtered to 6 mm resolution and both images closely resemble the reference image from the HR+ scanner with 6 mm resolution.

**Figure 6:**
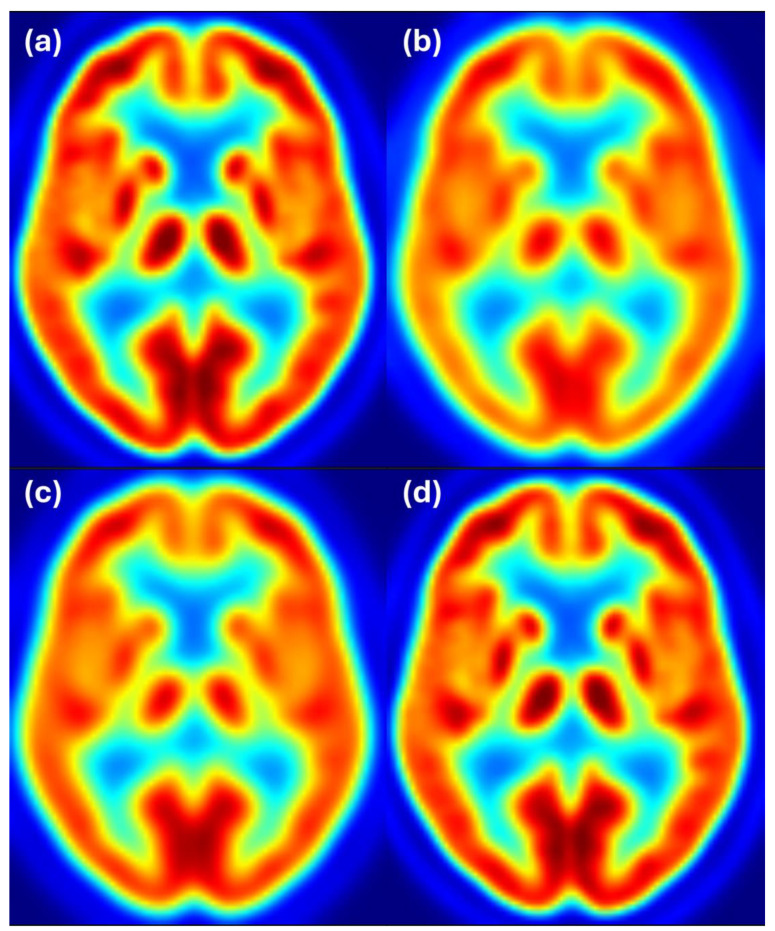
Harmonization results for the HR+ scanner matching the HRRT images filtered to 6 mm resolution. Image (**a**) shows the unfiltered HR+ image, (**b**) shows the HRRT image at the target resolution of 6 mm, (**c**) shows the HR+ image after filtering with the estimated filter, and (**d**) shows the HR+ image filtered to 6 mm resolution. Color scale indicates FDG uptake levels: red for high, green/yellow for medium, and blue for low uptake.

Figure 6 shows axial slices for 6 mm target resolution for HR+ matching HRRT resolution—the combination where the estimated filter considerably differs from the ground truth. We see that the HR+ image after filtering with the estimated filter follows the reference image at 6 mm resolution, while the HR+ and HRRT images at 6 mm resolution appear to have slightly different resolution. Color scale indicates FDG uptake levels: red for high, green/yellow for medium, and blue for low uptake.

**Figure 7:**
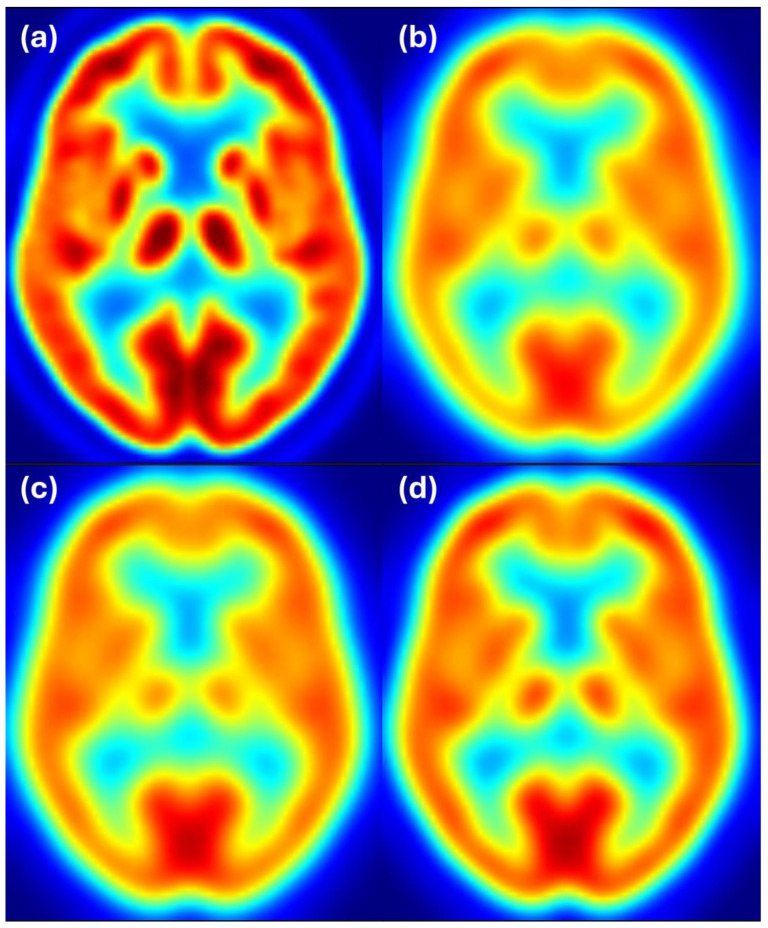
Harmonization results for the HR+ scanner matching the HRRT images filtered to 8 mm resolution. Image (**a**) shows the unfiltered HR+ image, (**b**) shows the HRRT image at the target resolution of 8 mm, (**c**) shows the HR+ image after filtering with the estimated filter, and (**d**) shows the HR+ image filtered to 8 mm resolution. Color scale indicates FDG uptake levels: red for high, green/yellow for medium, and blue for low uptake.

Figure 7 shows axial slices for the 8 mm target resolution for HR+ matching HRRT resolution. Also, here, the HR+ image after filtering with the estimated filter follows the reference image at 6 mm resolution, while the differences between the HR+ and HRRT images at 8 mm resolution are hardly noticeable. Color scale indicates FDG uptake levels: red for high, green/yellow for medium, and blue for low uptake.

## 4. Discussion

In this study, we developed a data-driven image-based harmonization protocol for FDG-PET brain images, aiming to determine the appropriate scanner-specific smoothing filters to achieve comparable image resolutions across different scanners. Unlike traditional approaches that utilize phantom scans for cross-scanner calibration, our method leverages the inherent characteristics of spatially normalized PET images.

Our results show that data-driven image-based harmonization can match the image resolution of datasets from two different scanners. Some scanner combinations had almost perfect matching (e.g., Discovery to HR+), while others had notable errors in filter estimation (e.g., HR+ to HRRT). There are some systematic discrepancies in the estimated Gaussian filter FWHM when matching different scanners. For example, when the HRRT is matched to other scanners, the estimated filter is consistently lower than the ground truth. Conversely, when other scanners are matched to the HRRT, the estimated filter is consistently higher than the ground truth. It is unclear why we observe this systemic discrepancy between the estimated Gaussian filter FWHM and the phantom-determined filter FWHM. One possible reason for this is the different noise properties of scanners, where the “noise” refers to all sources of stochastic variability in the measured signal, including photon statistics, detector performance, and reconstruction algorithms. The HRRT is an old scanner with very good resolution but tends to have a higher noise level [33], which might be the reason for that systemic discrepancy. However, the average image does not appear to be noisy, so the noise of the scanner should not have direct effect on the estimated filter. But it can have indirect effects. For example, noisier images can have less perfect spatial normalization, which could result in worse spatial resolution of the average image. Higher image noise may also cause higher error in the estimated scanner resolution from the phantom scans.

We observed that the discrepancy between the estimated Gaussian filter FWHM and the phantom-determined filter FWHM is lower when the target resolution is worse. For the target resolution of 10 mm FWHM, the estimated Gaussian filter FWHM is always within 1 mm of the phantom-determined filter FWHM. For the 8 mm FWHM target image resolution, the estimated Gaussian filter FWHM differs from the phantom-determined filter FWHM by up to 1.2 mm, while for the target resolution of 6 mm, the differences between the estimated Gaussian filter FWHM and the phantom-determined filter FWHM can be substantial—up to 3 mm. The reduced accuracy observed at the 6 mm target resolution may be due to the small size of the required Gaussian filters. When the intrinsic scanner resolution is already near the target resolution, only minimal additional filtering is needed, often less than 1 mm FWHM. Accurately estimating such narrow filters is challenging, as the effect on the image is subtle and easily masked by residual differences in images. When the target resolution is low, the image does not contain fine details and small differences in images are hidden.

The SSIM map as a function of XY and Z Gaussian filter FWHM (Figure 3) is smooth and does not exhibit local extrema. Therefore, optimization procedures are likely to converge reliably to the global minimum and unsuccessful parameter optimization is unlikely to be the source of errors in the estimated Gaussian filter FWHM. Based on the shape of the SSIM maps, we can expect the uncertainty of estimated filters in a range of 1 mm, which corresponds to the errors in the estimated filter FWHM in Figure 1 and Figure 2.

We did not observe substantial direction-specific differences in the results. Some differences obviously exist, as there are also some direction-dependent differences in the intrinsic resolution of the scanners. However, the SSIM map (Figure 3) shows that estimates of the XY and Z Gaussian filter FWHM are anti-correlated. This is evident from the shape of the level curves of the objective function near its minimum, which forms an ellipse rotated with respect to the coordinate axes. The orientation of this ellipse indicates a negative covariance between the estimated parameters, implying that an increase in one parameter tends to be compensated by a decrease in the other.

Similarly, we also did not observe substantial differences between the two subject groups. The minor differences are likely caused by random effects and the fact that the distributions of subjects among the different scanners were not the same for both subject groups.

Our method occupies a complementary space between some previous approaches. Like Jin et al. [31], we operate entirely on clinical images and do not rely on phantom data. However, unlike their empirical smoothing strategy, our method explicitly estimates the Gaussian filter required to harmonize one scanner’s resolution to match a reference. While our filter estimates may deviate more from phantom-based ground truth than the error in the estimated scanner resolution of Carbonell et al. [32], our goal is not to recover intrinsic scanner resolution per se, but rather to solve the practical problem of resolution matching. Therefore, the error in the estimated filter is not directly comparable to the error in the estimated intrinsic scanner resolution. This distinction is especially important in retrospective multicenter datasets, where scanner resolution is often unknown and varies due to acquisition or preprocessing differences. In such settings, even small uncertainties in estimated resolution may propagate into larger uncertainties in the derived harmonization filter, which our approach accounts for through scanner-specific filter estimation.

Unlike metric-based harmonization methods such as ComBat, which operate on extracted features or summary statistics, the proposed method works directly in image space. This distinction is not only technical but also conceptual. ComBat requires a predefined analysis pathway and is typically applied to specific scalar metrics derived from the images (e.g., regional SUV values or radiomic features). In contrast, our method harmonizes the spatial resolution of the entire image volume, making it suitable for a broad range of subsequent analyses, including voxelwise comparisons, statistical parametric mapping, or other image-based workflows. It thus provides greater flexibility in studies where the downstream analysis is not yet fixed or where multiple analyses are planned. While both approaches aim to reduce inter-scanner variability, they serve different use cases and may be seen as complementary rather than interchangeable.

Despite the promising results, our study has several limitations that should be acknowledged. This study did not account for the different noise levels of the scanners, particularly the HRRT scanner, which is known for its high spatial resolution but also has higher noise levels compared to other scanners. This inherent noise could contribute to the systematic discrepancies observed in the estimated Gaussian filter FWHM.

Spatial normalization introduces a known source of variability in image-based analyses. Although all images in this study were visually checked and found to be acceptably aligned, small residual mis-registrations cannot be avoided. However, since the harmonization procedure operates on averaged images, the effects of individual anatomical variability and minor registration errors are substantially reduced. In fact, from a signal-processing perspective, slight misalignment has a similar effect to mild image blurring. Therefore, its influence on the final estimation of smoothing parameters is comparable to that of additional low-level filtering. This makes the method relatively robust to small imperfections in spatial normalization, though future work could explore more precise registration techniques or multi-template approaches to further mitigate this source of error.

Additionally, this study did not aim to have an equal number of subjects for each scanner, resulting in the HRRT having the lowest number of subjects. This smaller sample size may have limited the noise reduction achieved through averaging. However, average images do not appear to be noisy, so the balanced number of subjects should not be critically important.

The discrepancies between the estimated and phantom-determined Gaussian filter FWHM were more pronounced at lower target resolutions (e.g., 6 mm FWHM). Therefore, our protocol may be less accurate for achieving finer resolutions and this limitation should be considered when using the proposed method for harmonization.

The ADNI preprocessing pipeline includes steps such as co-registration, averaging, and resampling, and aims to reduce inter-scanner variability. However, this process does not guarantee full harmonization of image resolution across scanner types. In this study, we relied on the filter widths provided by ADNI for harmonization to 8 mm resolution and used these to estimate the intrinsic resolution of each scanner under the assumption of quadratic combination of Gaussian kernels. While we did not explicitly quantify scanner-specific noise characteristics or residual biases introduced by preprocessing, our method is designed to operate on such preprocessed images and further reduce inter-scanner differences by data-driven estimation of resolution-matching filters. As such, it complements existing standardization procedures and addresses their limitations in retrospective multicenter studies.

It is important to note that spatial resolution in PET imaging is not constant across the transaxial field of view but typically degrades with increasing radial distance from the center. Both the ADNI-reported resolution values and our harmonization protocol rely on effective resolution estimates that do not explicitly capture this radial dependency. As a result, applying a uniform Gaussian filter across the image may not fully account for local resolution variability. However, this approach remains consistent with current harmonization practices, including those employed by the ADNI consortium, where a single global filter is used. Our method focuses on estimating scanner-specific global filter parameters to match the average spatial resolution between scanners without requiring phantom data. Future work may explore incorporating spatially varying resolution models or position-dependent filtering strategies to further improve harmonization accuracy.

Although the present study focused exclusively on FDG-PET brain images, the proposed harmonization approach may, in principle, be extended to other brain PET tracers such as amyloid or tau. The core assumption of the method—that spatially normalized average images reflect scanner-specific resolution properties—is not tracer-specific. However, differences in tracer distribution patterns, noise characteristics, or spatial uptake variability may influence the performance of the method. As such, the applicability of the protocol to other radiopharmaceuticals remains to be tested. Future studies are needed to assess whether additional adjustments or validation steps are required when applying this harmonization approach to alternative tracers.

During the initial phase of development of the harmonization protocol, several similarity metrics were evaluated, including mean squared error (MSE), peak signal-to-noise ratio (PSNR), normalized cross-correlation (NCC), and mutual information (MI). Among these, the Structural Similarity Index Metric (SSIM) provided the most robust and interpretable results for optimization. The MSE and PSNR were highly sensitive to global intensity scaling. The MI and NCC often provided similar results to the SSIM, but in some cases the results were far worse than for the SSIM. Therefore, the SSIM was selected as the similarity metric. Further research could focus on optimizing the similarity metric, including the metrics that are a combination of various elementary metrics mentioned above.

At present, the harmonization method is intended for use on group-averaged images. Averaging across subjects suppresses random noise and allows for stable estimation of scanner-specific smoothing parameters using the SSIM-based optimization framework. While application to single-subject images is conceptually possible, the method may be too sensitive to noise in that context, leading to unstable or biased filter estimates. Extending the approach to individual-level data would require advanced noise modeling or regularization techniques to ensure reliable performance and remains an interesting avenue for future research.

## 5. Conclusions

Our data-driven image-based harmonization protocol provides an effective method for harmonizing FDG-PET brain images across multiple scanners when the phantom scans are not available. The protocol demonstrates good and consistent performance across different scanners when the target resolution is not too close to intrinsic scanner resolution. As such, it offers a unique solution for scanner harmonization in various retrospective studies where phantom scans are limited or non-existent.

## Figures and Tables

**Table 1:** Selected scanner models, their effective resolution, and the number of AD and CN subjects selected for each scanner. Although some scanner groups include multiple hardware models, these were grouped together based on reconstruction-specific resolution data determined from phantom scans.

Scanner	Resolution FWHM [mm]		Number of Subjects	
	XY	Z	AD	CN
HRRT (Siemens)	4.5	4.5	12	16
Biograph (Siemens; models HiRes/mCT/1093/1094/1080)	5.5	5.5	32	37
Discovery (General Electric; models 600, 690, RX, STE)	5.5	6.0	35	38
HR+ (Siemens)	6.0	6.0	41	29

## Data Availability

The data that support the findings of this study are available upon reasonable request from the authors.
